# Application of digital tools and artificial intelligence in the Myasthenia Gravis Core Examination

**DOI:** 10.3389/fneur.2024.1474884

**Published:** 2024-12-04

**Authors:** Marc Garbey, Quentin Lesport, Helen Girma, Gülşen Öztosun, Mohammed Abu-Rub, Amanda C. Guidon, Vern Juel, Richard J. Nowak, Betty Soliven, Inmaculada Aban, Henry J. Kaminski

**Affiliations:** ^1^Department of Surgery, School of Medicine & Health Sciences, George Washington University, Washington, DC, United States; ^2^Care Constitution Corp., Houston, TX, United States; ^3^LaSIE, UMR-CNRS 7356, University of La Rochelle, La Rochelle, France; ^4^Department of Neurology & Rehabilitation Medicine, School of Medicine & Health Sciences, George Washington University, Washington, DC, United States; ^5^Division of Neuromuscular Medicine, Department of Neurology, Massachusetts General Hospital, Harvard Medical School, Boston, MA, United States; ^6^Department of Neurology, Duke University School of Medicine, Durham, NC, United States; ^7^Department of Neurology, Yale School of Medicine, New Haven, CT, United States; ^8^Department of Neurology, The University of Chicago, Chicago, IL, United States; ^9^Department of Biostatistics, University of Alabama at Birmingham, Birmingham, AL, United States

**Keywords:** telehealth, myasthenia gravis, ptosis, diplopia, deep learning, computer vision, neurological disease, clinical trial

## Abstract

**Background:**

Advances in video image analysis and artificial intelligence provide opportunities to transform how patients are evaluated. In this study, we assessed the ability to quantify Zoom video recordings of a standardized neurological examination— the Myasthenia Gravis Core Examination (MG-CE)—designed for telemedicine evaluations.

**Methods:**

We used Zoom (Zoom Video Communications) videos of patients with myasthenia gravis (MG) who underwent the MG-CE. Computer vision, in combination with artificial intelligence methods, was used to develop algorithms to analyze the videos, with a focus on eye and body motions. To assess the examinations involving vocalization, signal processing methods, such as natural language processing (NLP), were developed. A series of algorithms were developed to automatically compute the metrics of the MG-CE.

**Results:**

A total of 51 patients with MG were assessed, with videos recorded twice on separate days, while 15 control subjects were evaluated once. We successfully quantified the positions of the lids, eyes, and arms and developed respiratory metrics based on breath counts. The cheek puff exercise was found to have limited value for quantification. Technical limitations included variations in illumination, bandwidth, and the fact that the recording was conducted from the examiner’s side rather than the patient’s side.

**Conclusion:**

Several aspects of the MG-CE can be quantified to produce continuous measurements using standard Zoom video recordings. Further development of the technology will enable trained non-physician healthcare providers to conduct precise examinations of patients with MG outside of conventional clinical settings, including for the purpose of clinical trials.

## 1 Introduction

Telemedicine and multi-modal patient monitoring technologies are aimed at revolutionizing conventional clinical care and enhancing the efficiency of clinical trials (1–3). These advancements hold particular promise for patients facing barriers to accessing healthcare, such as those with disabilities, limited economic resources, and a lack of caregiver support. Furthermore, these obstacles may prevent patients from participating in clinical research studies, which require frequent monitoring visits. Individuals with rare diseases may face more pronounced challenges due to the scarcity of care centers and clinical trials specifically for these conditions (4). Enhancing the inclusivity and efficiency of clinical trial processes is critically important to advance therapeutic development (5).

Inter-rater variability in clinical trial outcome measures is well recognized. This variation can result from ambiguity in the metrics used, as well as from the differences in how evaluators perform or assess a specific outcome measure (6–8). Video-based examinations using accessible technology offer opportunities for synchronous or post-hoc quantitative analyses. Improved outcome measures and clinical care approaches are particularly needed for myasthenia gravis (MG), given its rarity, propensity for fluctuation, and the benefit from extensive disease-specific monitoring by subspecialists (7–9).

A standardized examination for MG, specifically tailored for telemedicine use, was developed at the start of the COVID-19 pandemic (10). This assessment, known as the MG-Core Examination (MG-CE), is based on the traditional neuromuscular examination performed in clinics and insights gained from outcome measures used in clinical trials for MG. We took advantage of a bank of recorded video sessions to create algorithms aimed at quantifying various aspects of the MG-CE (11, 12). In this study, we aimed to apply and refine our algorithms using a large cohort of MG patients and a diverse control sample. Our approach consistently identified key examination metrics, and we recognized aspects of the MG-CE that are not reliable. Our methods have the potential to enhance the way the MG-CE is conducted and to provide quantitative assessments that were previously unattainable.

## 2 Methods

### 2.1 Participants and video recording

Participants were recruited to undergo standardized examinations via telemedicine to assess the performance of the MG-CE (NCT05917184) (10). These video recordings were not created specifically for this study’s purpose and accurately reflect a standard telemedicine visit. We accessed the recordings of participants who had undergone the examinations twice within 7 days, except for one patient who had a gap of 39 days between the videos. Each participant was evaluated by the same neurologist who holds board certification in neuromuscular medicine. Control participants were selected based on having no self-reported physical limitations and a score of zero on the MG Activities of Daily Living (13). The MG-CE was performed once by a board-certified neurologist. A total of 51 patients with MG and 15 controls participated in the study (Supplementary material).

All participants provided written consent. The patient study was approved by the central institutional review board of MGNet at Duke University and the George Washington University institutional review board. All patients exhibited clinical characteristics of MG, which were confirmed by elevated serum autoantibody levels or electrophysiological findings. The control study was approved by the George Washington University institutional review board.

The present dataset allowed us to further train our algorithms (11, 12) using a broad spectrum of computer vision and signal analysis tools in combination with artificial intelligence (AI) methods. We systematically used an AI transcription tool, AssemblyAI (San Francisco, CA), and standard natural language processing (NLP) techniques to time stamp the patient reports, such as descriptions of double vision and counting exercises. Details of the methods used for each examination and their technical limitations are presented in the Supplementary material.

## 3 Results

### 3.1 Quality review

We found great variability in the pixel count of the patient images and lighting conditions across recordings. The number of frames per second was 25, with the exception of 5 videos that were recorded at 30 frames per second. To maintain the quality of data acquisition, a video was removed from the analysis if any of the following conditions were met: (1) the individual was positioned too close to the camera to capture a clear view of the region of interest, (2) inadequate illumination or lack of contrast resulted in insufficient pixel count, or (3) the audio volume was too low to allow effective speech evaluation. Supplementary Table S2 lists the number of videos excluded from the analysis for each metric, while the Supplementary material provides the reasons for their elimination.

### 3.2 Ptosis evaluation

**Figure 1:**
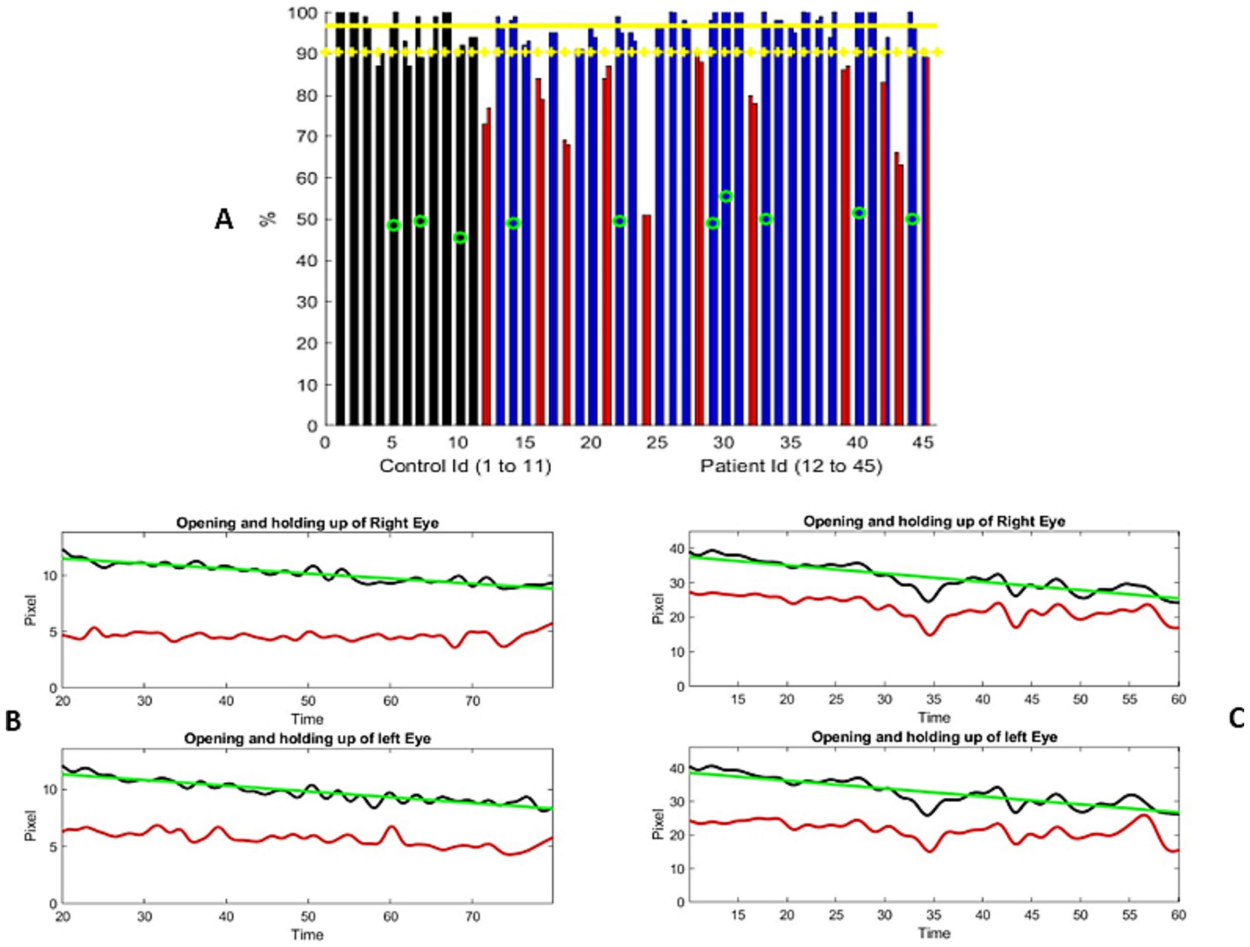
**(A)** Distribution of the percentage of change from the maximum separation of the upper and lower eyelids during the examination. The controls are represented using two adjacent black bars, one for each eye. Similarly, the patient results are shown in blue or red bars. The most severe ptosis cases from the two evaluations are presented here. Red columns represent the participants who were significantly different from the controls (one standard deviation from the mean). Green circles represent the individuals older than 70 years. Yellow horizontal lines represent the mean metric output for the control, with the mean minus one standard deviation serving as the threshold to mark the presence of ptosis. **(B,C)** Two examples of the variation in the distance between the upper lid and lower lid (black curve) and the distance from the bottom of the iris to the lower lid (red curve), measured in pixels, over the course of the exercise. The horizontal axis represents the time elapsed during the exercise, in seconds. Figure **(B)** shows a progressive, linear, and continuous eyelid fatigue well fitted by a linear square (green curve), while **(C)** shows the measurements of a participant struggling to keep the eyes open. It is important to note that the number of pixels representing the eye opening differs between these two participants, which reflects the dataset, where the distance between the participant and the camera was not standardized. The participant in **(B)** was assessed to have moderate ptosis, while the participant in **(C)** was graded from no ptosis to mild and moderate ptosis.

A sample of 540 images from the video dataset of the controls was manually evaluated so that the obtained anatomical landmarks were within 2 pixels. We were able to compute the distance from the upper lid to the bottom of the iris and the distance from the upper lid to the lower lid. Figure 1 shows the results of 11 out of 14 controls. The recordings for the ptosis assessment had pixel resolutions ranging from 7 to 28. We found that one-third of the videos had poor resolution. The four-fold difference between the lowest and highest resolution of the videos was largely due to the variations in the distance between the participant and the camera. The aspect ratio of the eye width versus height varied from 33 to 55 percent, which contributed to variability in the video acquisition. The noise in the numerical output was at most 4% after filtering (11). No control exhibited greater than 4%variation, except for those older than 70 years. Therefore, our use of the 70-year-old boundary needs to be evaluated.

A total of 34 patient videos were analyzed (Figure 1A). We identified two patterns of lid fatigue. One pattern was a smooth, linear drop in the upper lid, approximated by the negative slope obtained through linear square fitting. The second pattern exhibited a more chaotic behavior, where the patient appeared to struggle to maintain upgaze (Figures 1B,C). Of the 34 patients, 11 exhibited lid fatigue. As we had established a 4% variation for the controls, we did not expect false-positive results for patients above that threshold. A total of three patients progressively tilted their heads backward to compensate for ptosis, which compromised measurement precision. This was detected by the change in the vertical dimension from the chin to the top of the head. We were able to quantitatively and continuously monitor lid fatigue.

### 3.3 Ocular alignment

**Figure 2:**
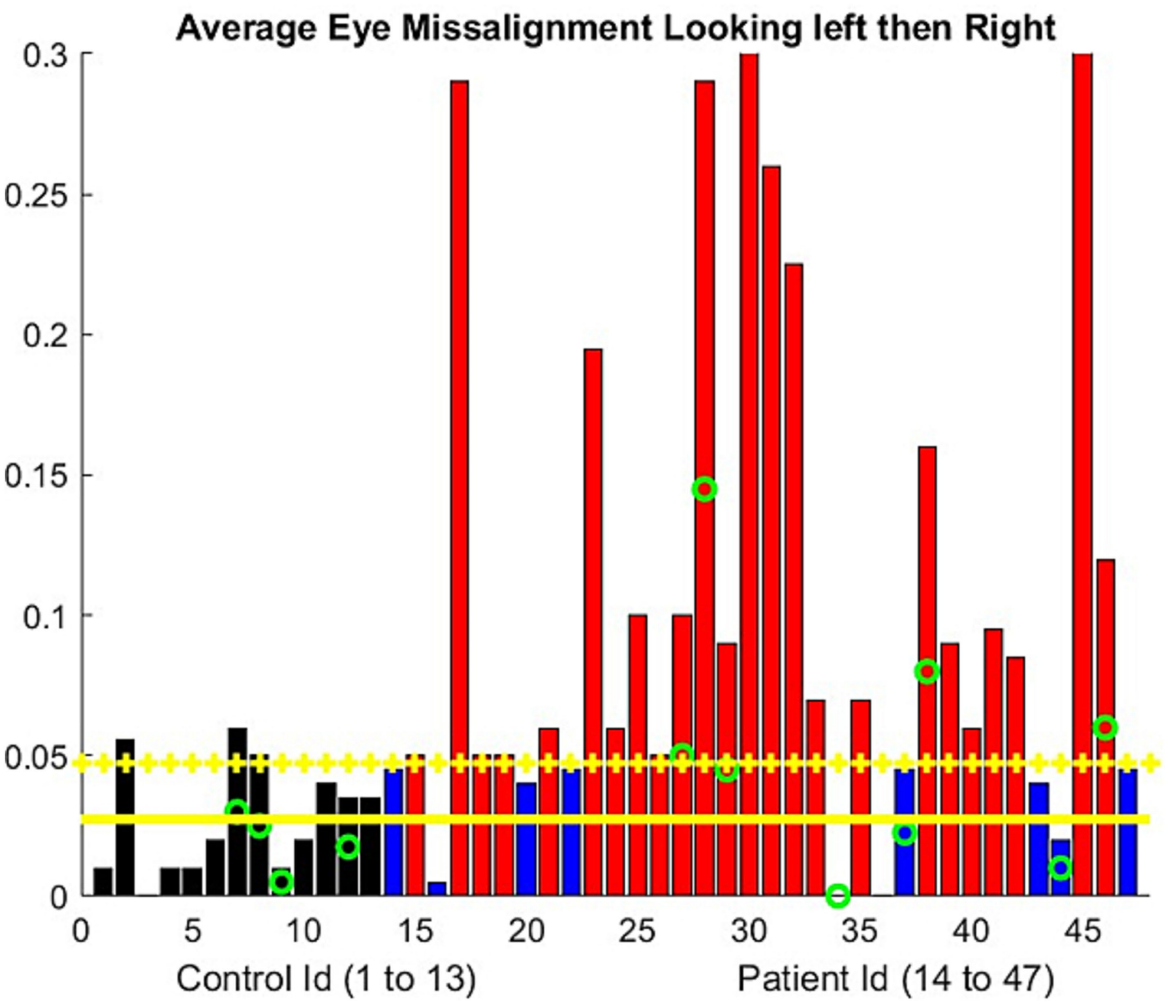
Ocular alignment was assessed by the difference in the barycentric coordinate (0 = no misalignment). The progressive misalignment between both eyes used the least squares approximation of the barycentric coordinate change during the exercise. The controls are represented using a black bar. The patient results are shown using blue bars if there is no significant misalignment developed during the examination and red bars if misalignment was present. The green circle represents the participants older than 70 years. Yellow horizontal lines represent the mean metric output for the control, with the mean plus one standard deviation serving as the threshold to mark the presence of eye misalignment.

Figure 2 shows the results of the barycentric coordinate determination of the visible iris boundary for 13 controls. The two instances of failure were related to participants turning their heads or poor lighting conditions. We observed that eye opening was marginally smaller for the controls during this test compared to the ptosis assessment. Ocular alignment could be determined within 5%. Therefore, a 5% error was the threshold above which ocular misalignment could be estimated.

For the patients, the average vertical opening of the lids during the eccentric gaze in the diplopia test was smaller compared to the controls. We obtained a ratio of 0.63, with a standard deviation of 0.17. Consequently, the number of pixels available to assess the iris position was lower than that during the ptosis evaluation. Ocular misalignment was computed only along the horizontal axis (12) In two patients, our algorithm failed because the misalignment was primarily in the vertical direction. A few patients demonstrated a stable alignment of their eyes based on the barycentric coordinate we computed but still reported experiencing double vision. However, others had double vision without any noticeable misalignment. In fact, the resolution of the videos usually does not allow the human examiner to assess ocular misalignment. The NLP algorithm allows precise identification of the timestamp for reported double vision. We observed (Figure 2) that approximately half of the patients experienced a drift in ocular alignment during the test.

### 3.4 Arm extension

**Figure 3:**
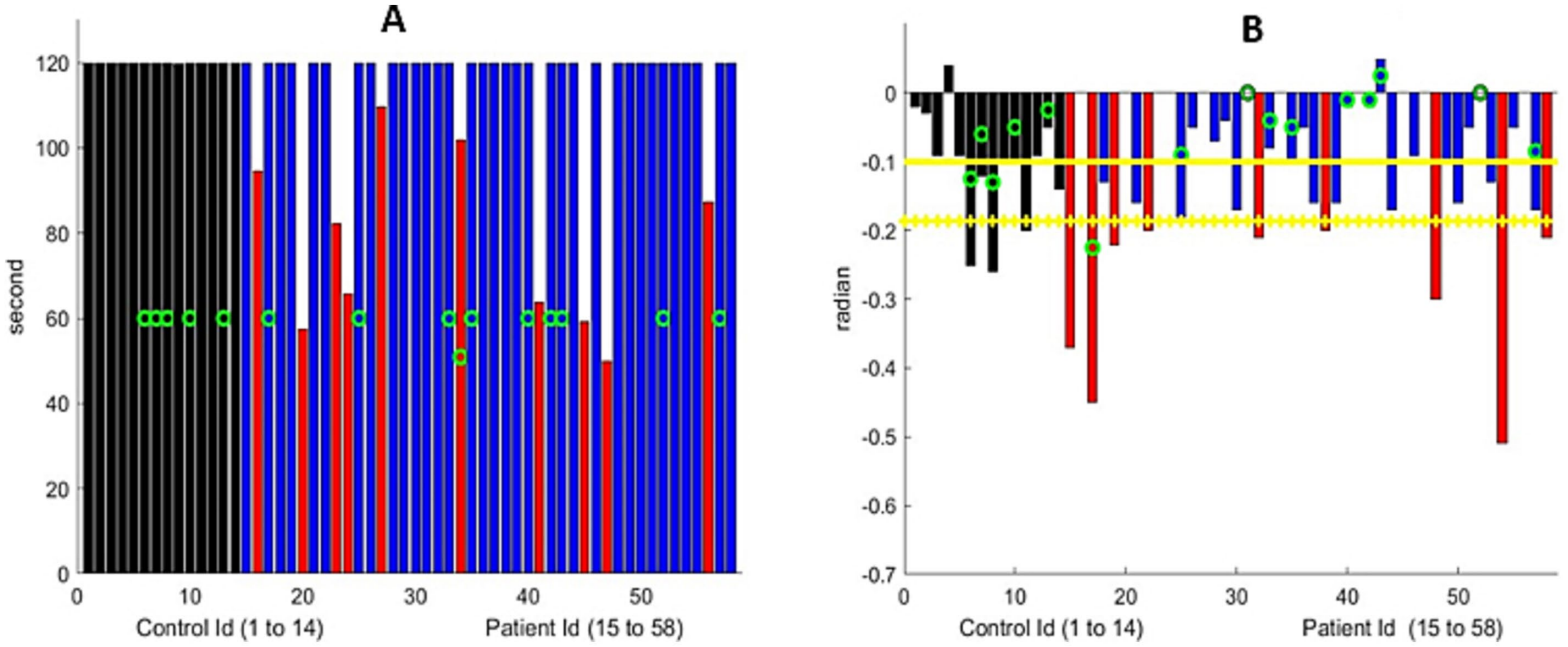
**(A)** Maximum arm extension time. All controls reached the 120-s limit. Nine MG patients (marked with a red bar) were unable to do so. The green circle represents the patients older than 70 years. **(B)** Linear drift of the arm extension, in radians, for the participants who could extend their arms for 120 s. The black bar represents the control group, and the blue bar represents MG patients who were similar to the controls. The red bar represents the MG patients with significantly greater drift. We show the maximum drift for both arms at both visits for MG patients. The green circle represents the participants older than 70 years. Yellow horizontal lines represent the mean metric output for the control group, with the mean minus one standard deviation serving as the threshold to mark the presence of shoulder muscular weakness.

Figure 3 and Supplementary Figure S1 illustrate the elapsed time during which the participant could maintain shoulder abduction to approximately 90 degrees with the elbows fully extended. Our algorithm measured the angle formed by the arm and torso, as well as the vertical variation. Our assessment required that the patient remain seated during the exercise and that the camera image show both arms; however, an examiner can evaluate the exercise with only a partial view of the arms. The algorithm computed the total time the participant maintained shoulder abduction up to a 2-min duration, as well as the slope of the decay of the angle formed by each arm and the vertical arm position during the elapsed time.

Despite the variation in the initial arm positions among the participants, the algorithm successfully analyzed all the controls, except for one participant who had an obstructed view of the arms. The average downward drift during the test was −0.05 radians for those younger than 70 years of age and −0.15 radians for those older than 70. Therefore, subtracting the standard deviation to detect fatigue provides a threshold of −0.12. In other words, any slope less negative than this would indicate fatigue for patients younger than 70 years and −0.24 for those older than 70.

Of 44 patients, 10 were unable to maintain their arms extended for 2 min, while an additional 9 individuals experienced significant drift (Figure 3 and Supplement Figure S2). The decay of the angle was, in the first approximation, linear. In other words, the drift of the arms from the horizontal was a continuous (linear) process that starts at time zero. We manually assessed that both metrics were computed correctly with great accuracy, provided that the torso, head, and both arms were within the frame of the video. In comparison to the formal MG-CE scoring, arm drift was not assessed, and only the ability to hold for 2 min was measured. Quantifying drift through examiner review of the video is difficult and may be impossible. However, the digital algorithm can identify abnormalities.

### 3.5 Sit-to-stand exercise

**Figure 4:**
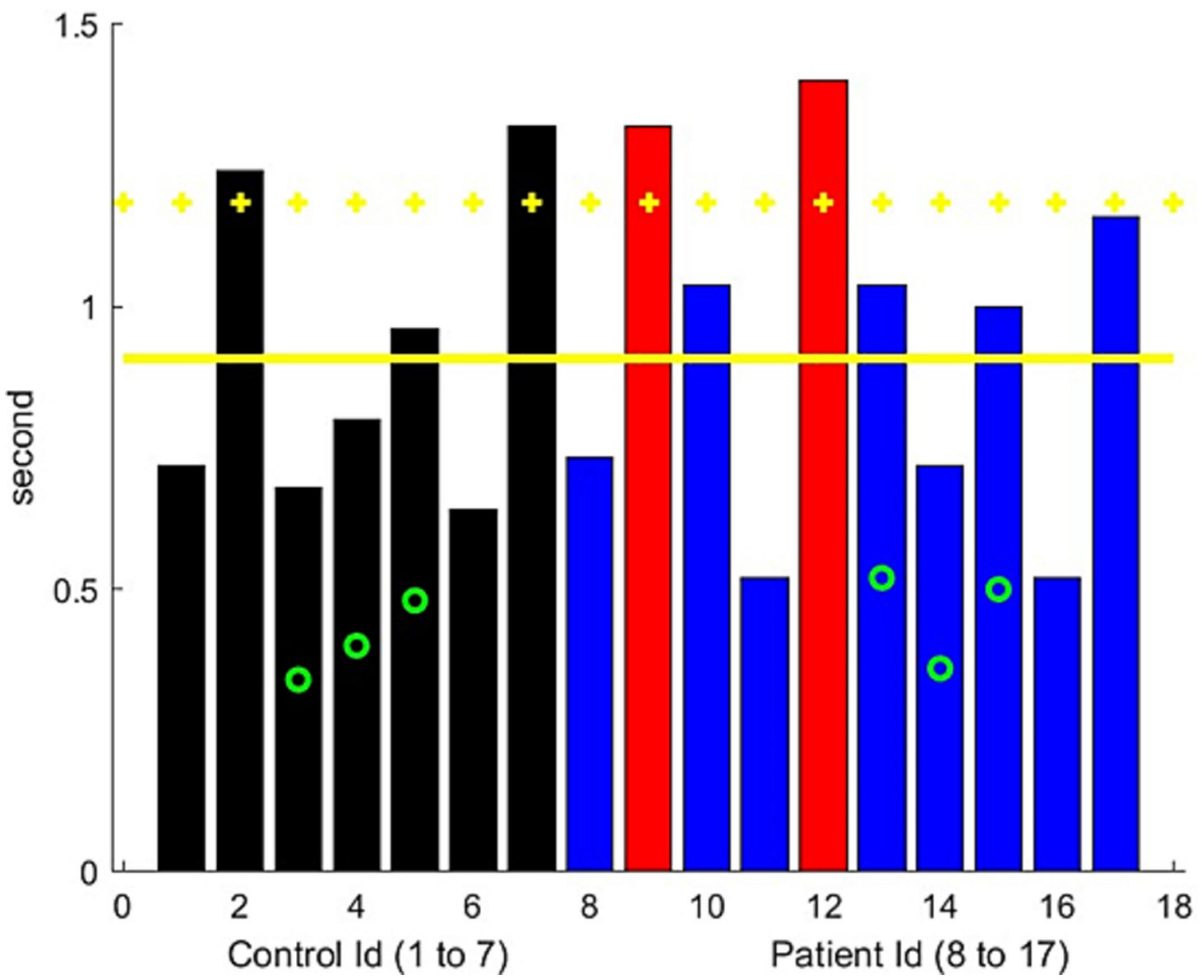
Ascending time for the sit-to-stand exercise. The control results are represented in black, while the results of the MG patients are represented in blue, with red bars indicating significantly worse performance than controls. The green circle represents the patients older than 70 years. Yellow horizontal lines represent the mean metric output for the control group, with the mean plus one standard deviation serving as the threshold to mark the presence of leg muscular weakness.

We evaluated the elapsed time in the ascending phase for the participants who could stand with arms crossed (Figure 4). All controls were able to stand with their arms crossed or uncrossed. We discarded five control videos and nearly half of the patient videos because either the head or the hips were not visible. An additional three participants had only a partial view of the head upon standing. The automatic identification of the sit-to-stand time was hindered by the time lag of the algorithm and the limited number of frames per second in the video, resulting in poor accuracy (Figure 4). We manually assessed all entries and found an error of less than20% for 7 controls and 10 patients in the videos with a complete view of the body during the test. This error was 40% for the entire dataset, including 10 controls and 28 patients, for the videos with a partial view of the head in the standing position. We were easily able to automatically detect when a patient could not stand.

### 3.6 Count-to-50 exercise

Using NLP, the identification of the soundtrack time windows corresponding to the exercise was successful with high accuracy. Dysarthria was not observed in the controls by the neurologist examiner. We first computed the time length of the count-to-50 exercise for each patient and compared the elapsed times of the two evaluations. We observed a relatively good consistency in the times for the same patient across two different visits, but there was significant variability among the patients in the frequency of counting, with the elapsed times ranging from 0.5 to 1.5 s.

**Figure 5:**
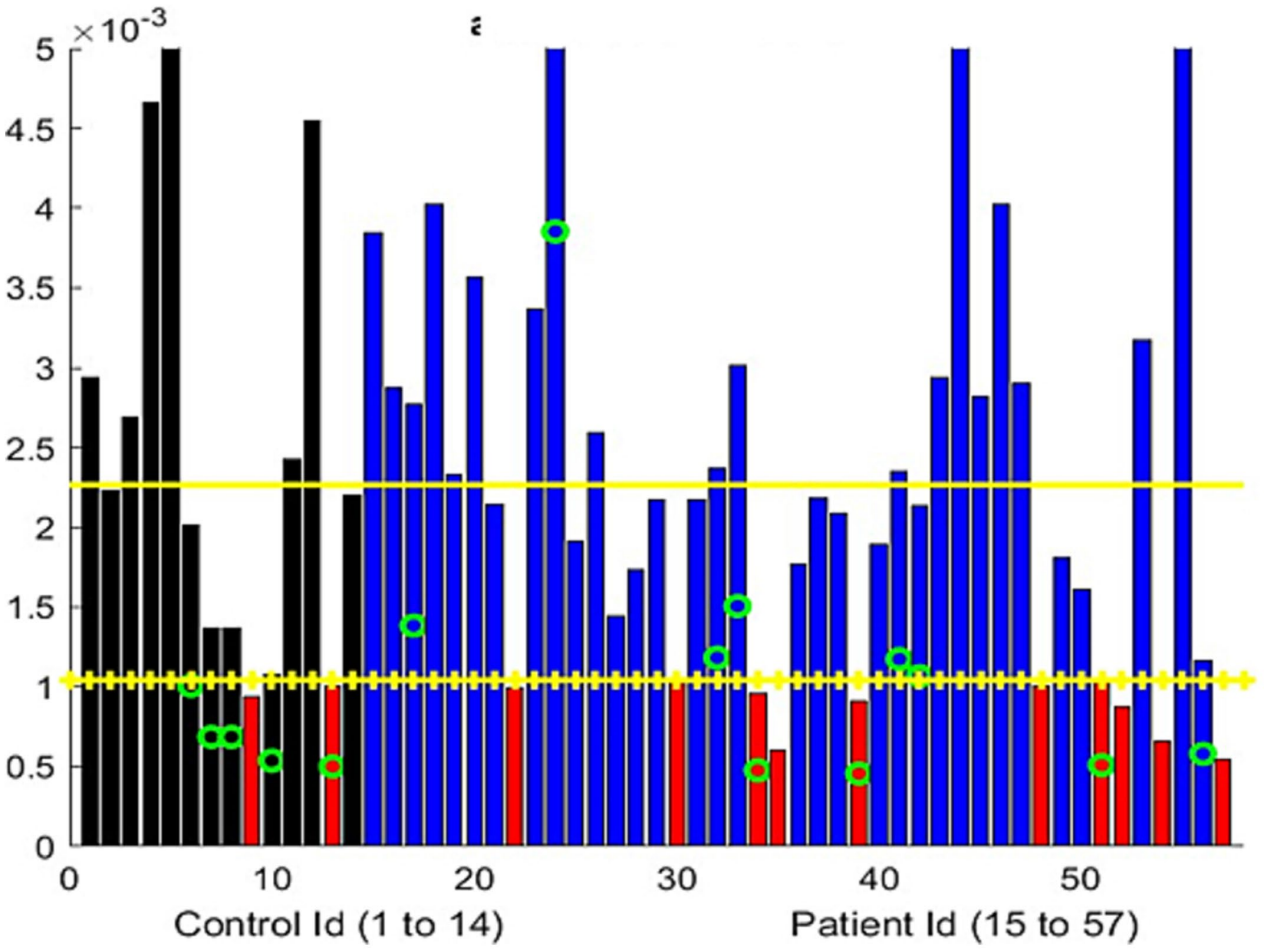
Average variation of the acceleration (normalized distance/s square) of the vertical movement of the upper lip during the count-to-50 exercise. Black bars represent the controls, blue bars represent the MG patients, and red bars indicate significantly worse performance than the controls. The green circle represents the participants older than 70 years. Yellow horizontal lines represent the mean metric output for the control group, with the mean minus one standard deviation serving as the threshold to mark the presence of lip/facial muscular weakness.

We were able to compute the dynamic motions of the lips and mouth. The acceleration of the vertical component of mouth motions could indicate muscle weakness in the lips and, to some extent, in the cheek and jaw muscles (14). Mouth motions in the controls over 70 years of age were generally slower. Of the 49 patients, 4 were rated as having dysarthria by the physicians, while 5 were rated as having possible dysarthria, based on one of two evaluators identifying the point at which dysarthria appeared (publication in preparation). A low acceleration value was noted for all these patients (Figure 5). However, weakness in the vocal folds, pharyngeal muscles, tongue, and soft palate muscles can also cause dysarthria. This could be best assessed through sound analysis (15–17).

### 3.7 Single-breath-count

The NLP was able to determine the total time for test performance and the number the participant counted to. All controls under 70 years of age, except one with a large body habitus, had no difficulty counting to 30. We computed the elapsed time for counting in one breath, as opposed to the time taken to count to the last number (Supplementary Figure S1). The participants were not asked to count at a specific rate, as per the MG-CE instructions, and maintaining a particular pace was difficult. We observed that age impacted the participants’ performance. However, this measure was likely an underestimation because the young controls may have counted for a longer period.

The total time counted varied between visits for individual participants (Supplementary Figure S2). The instructions stated that participants should count as high as possible in one breath, but no attempt at standardization was recommended. Therefore, it was difficult to use this exercise to assess respiratory function. The variation in patient responses between the visits could have been caused by MG, prior activities, or the time elapsed since the last dose of pyridostigmine. We were unable to correlate speech features from the count-to-50 exercise with those from the single-breath-count exercise, as we did in our previous study (11). This discrepancy is likely related to the greater variation across study sites.

### 3.8 Cheek puff and tongue protrusion

Both evaluations proved to be the most difficult to segment using computer vision. We were able to assess whether the participants were able to form a tight “O” seal with the lips when asked to fill their cheeks with air. We computed the pattern/amount of cheek deformation in both the cheek puff and tongue-to-cheek maneuvers, as described previously (11). However, the variations in anatomy prevented us from establishing baseline values to compare the control and MG groups.

## 4 Discussion

In this study, we demonstrated that asynchronous quantitative analyses of the MG-CE are feasible. For the majority of the exam components, our algorithm distinguished significant differences between the MG and control groups. In contrast to concerns regarding the inferiority of telemedicine examinations compared to in-person assessments (1, 18), we found that significant potential exists for quantifying the neuromuscular examination through objective analytical methods.

Despite the inherent technological limitations of telemedicine and the videos *not* being originally recorded or optimized for our analyses, a significant majority could still be utilized to derive continuous measures of fatigue in MG patients. The Zoom video usability was directly proportional to the pixel resolution, uniformity of recording, and optimization of the patient’s lighting and environment. Telehealth offers a unique opportunity to leverage digital technology and AI (3, 19, 20) as all communication is in digital form and can be archived, revisited, and analyzed using improving algorithms. The manual work involved in this process was non-trivial and required human review of 50 h of videos. This is typical of rigorously developed AI applications, which require significant human input initially to ensure accurate algorithm development (21). Digital processing supplemented by AI can make the process more automatic, efficient, and consistent as we continue to optimize the technology.

Our AI algorithms allowed for the quantification of the ocular examination and offered the opportunity to perform objective evaluations, either via telemedicine or in person, by trained clinic staff for routine clinical care or by research coordinators in the context of clinical trials. Our analytics allowed for an estimate of the marginal reflex (the distance from the light reflection on the pupil to the upper lid) from the pre-recorded videos, provided that the lighting conditions and pixel resolution were sufficient to see the pupil clearly in relation to the upper lid position. Consistent with clinical practice, we did not always detect ocular misalignment, an objective measure, when the patients reported double vision. Specialized eye movement recordings or ophthalmological evaluations, which are not commonly performed by neurologists, are required to definitively assess ocular misalignment. Patients may also exhibit central adaptation to ignore the false image or may have vision impairment in one eye, which limits binocular diplopia. Our algorithm did not assess vertical misalignment, which could have resulted in double vision. MG patients exhibited a linear drift or chaotic instability of the horizontal eye position, most likely indicative of neuromuscular transmission failure (22). The majority of the patients demonstrated fatigable ptosis while maintaining a lateral eye position. This made the automatic detection of the anatomical landmarks for measuring ocular alignment more challenging in MG patients.

We were able to quantify the extended arm position by the elapsed time of the abduction and downward drift. Drift represents a continuous linear process that can be challenging for an examiner to track, whereas the instability in the arm position can persist until patients suddenly drop their arms. These observations may reflect specific aspects of neuromuscular transmission fatigue (23). The drift suggests a gradual reduction in the number of active muscle fibers capable of generating sufficient force, while the abrupt drop indicates the simultaneous loss of numerous fibers responsible for maintaining the arm position. Both physicians and patients recognize these distinct phenomena. Central or musculoskeletal factors may influence these results (24, 25).

Standing up from a seated position is a complicated movement. Weakness, sensory deficits, pain, and multiple other factors, including compensatory adjustments, may influence the movement. The variations in the test performance, most notably due to the patient’s environment and the examiner’s viewing position, further complicated our assessment. This exercise holds the greatest potential for optimization to achieve a clinically useful score, especially given the importance of rising from a seated position as an indicator of overall function (26).

We successfully utilized the count-to-50 and single-breath-count exercises to develop measures that could not be assessed by an examiner simply by viewing the videos. No reliable quantification of the deformation of the cheek could be established for the cheek puff task. The anatomical variation among the participants further contributed to our inability to derive a reliable assessment. The cheek puff and tongue-in-cheek exercises were not suitable for developing an assessment method. We question the utility of these tasks in telehealth evaluation since the examiner cannot touch the cheek to evaluate muscle strength.

Despite the limitations of the count-to-50 and single-breath-count exercises, we were able to derive assessments that were amenable to quantification, albeit in a manner different from the exam’s original intent. The lip and jaw movements could be evaluated with high accuracy and consistency across the two videos. Abnormalities were identified in the patients who were assessed to have dysarthria by the physician examiners. The single-breath-count exercise is often considered to be a good bedside test for assessing respiratory function; however, formal evaluations indicate only mild to moderate correlation with respiratory parameters in patients with less severe weakness (27, 28). We found significant variation in the counting speed among the participants, and we also found that the elapsed time was a more consistent metric.

The process of digitalization and its utilization of AI introduces new dimensions beyond the scope of human perception. Further investigations will define the added value of the increased accuracy offered by digital metrics compared to traditional observations, as well as the utility of incorporating these new metrics into clinical trials and practice. The digital algorithm outputs require an additional step to convert numerical data into a meaningful score. This process is similar to converting a laboratory test result into a disease progression score, for example, using a CD4 count to assess HIV infection (29). Our hypothesis is that digital processing inherently reduces ambiguity and hidden assumptions in protocol tasks, which may potentially enhance the quality of scoring and provide new metrics to model nervous system function (30). Furthermore, we envision our digital framework for conducting the MG-CE examination as an opportunity to (i) enhance physician training before clinical studies, (ii) fully leverage a dataset accumulated during a clinical trial with minimal human effort for subsequent analysis, and (iii) facilitate an agile approach to clinical trials that enables real-time examination of data to identify and address potential gaps and errors in data acquisition as early as possible.

## Data Availability

The raw data supporting the conclusions of this article will be made available by the authors, without undue reservation.

## References

[1] Zeiler SR Abshire Saylor M Chao A Bahouth M. Telemedicine Services for the Delivery of specialty home-based neurological care. Telemed J E Health. (2022) 29:1088–95. doi: 10.1089/tmj.2022.0242 36450111

[2] de Rezende DRB Neto IA Iunes DH Carvalho LC. Analysis of the effectiveness of remote intervention of patients affected by chronic diseases: a systematic review and meta-analysis. J Med Access. (2023) 7:27550834231197316. doi: 10.1177/27550834231197316, PMID: 37781504 PMC10540568

[3] Javed I Diana Carolina CJ Pallavi M Sachin S Sarah H Thanmai R. Reimagining healthcare: unleashing the power of artificial intelligence in medicine. Cureus. (2023) 15:e44658. doi: 10.7759/cureus.44658 37799217 PMC10549955

[4] Lumsden JM Urv TK. The rare diseases clinical research network: a model for clinical trial readiness. Ther Adv Rare Dis. (2023) 4:26330040231219272. doi: 10.1177/26330040231219272, PMID: 38152157 PMC10752072

[5] Fogel DB. Factors associated with clinical trials that fail and opportunities for improving the likelihood of success: a review. Contemp Clin Trials Commun. (2018) 11:156–64. doi: 10.1016/j.conctc.2018.08.001, PMID: 30112460 PMC6092479

[6] Coster WJ. Making the best match: selecting outcome measures for clinical trials and outcome studies. Am J Occup Ther. (2013) 67:162–70. doi: 10.5014/ajot.2013.006015, PMID: 23433270 PMC3628620

[7] Guptill JT Benatar M Granit V Habib AA Howard JF Jr Barnett-Tapia C. Addressing outcome measure variability in myasthenia gravis clinical trials. Neurology. (2023) 101:442–51. doi: 10.1212/WNL.0000000000207278, PMID: 37076302 PMC10491448

[8] Benatar M Cutter G Kaminski HJ. The best and worst of times in therapy development for myasthenia gravis. Muscle Nerve. (2023) 67:12–6. doi: 10.1002/mus.27742, PMID: 36321730 PMC9780175

[9] Benatar M Sanders DB Burns TM Cutter GR Guptill JT Baggi F. Recommendations for myasthenia gravis clinical trials. Muscle Nerve. (2012) 45:909–17. doi: 10.1002/mus.23330 22581550

[10] Guidon AC Muppidi S Nowak RJ Guptill JT Hehir MK Ruzhansky K. Telemedicine visits in myasthenia gravis: expert guidance and the myasthenia gravis Core exam (MG-CE). Muscle Nerve. (2021) 64:270–6. doi: 10.1002/mus.27260, PMID: 33959997 PMC9057373

[11] Garbey M Joerger G Lesport Q Girma H McNett S Abu-Rub M. A digital telehealth system to compute the myasthenia gravis Core examination metrics. JMIR Neurotechnol. (2023) 2:e43387. doi: 10.2196/43387, PMID: 37435094 PMC10334459

[12] Lesport Q Joerger G Kaminski HJ Girma H McNett S Abu-Rub M. Eye segmentation method for telehealth: application to the myasthenia gravis physical examination. Sensors. (2023) 23:7744. doi: 10.3390/s23187744, PMID: 37765800 PMC10536520

[13] Muppidi S Wolfe GI Conaway M Burns TM Mg C. Mg-Qol15 study G. MG-ADL: still a relevant outcome measure. Muscle Nerve. (2011) 44:727–31. doi: 10.1002/mus.22140, PMID: 22006686

[14] Mefferd AS Lai A Bagnato F. A first investigation of tongue, lip, and jaw movements in persons with dysarthria due to multiple sclerosis. Mult Scler Relat Disord. (2019) 27:188–94. doi: 10.1016/j.msard.2018.10.116, PMID: 30399501 PMC6333529

[15] Vogel AP Fletcher J Snyder PJ Fredrickson A Maruff P. Reliability, stability, and sensitivity to change and impairment in acoustic measures of timing and frequency. J Voice. (2011) 25:137–49. doi: 10.1016/j.jvoice.2009.09.003, PMID: 20171828

[16] Ljitona T Soraghan J Lowit A Di-Caterina G Yue H. Automatic detection of speech disorder in dysarthria using extended speech feature extraction and neural networks classification. IET 3rd International Conference on Intelligent Signal Processing (2017). London: IET: 1–6.

[17] Spangler T Vinodchandran N Samal A Green J. Fractal features for automatic detection of dysarthria. International Conference on Biomedical & Health Informatics (BHI). London: IET, (2017): 437–440.

[18] Giannotta M Petrelli C Pini A. Telemedicine applied to neuromuscular disorders: focus on the COVID-19 pandemic era. Acta Myol. (2022) 41:30–6. 35465343 10.36185/2532-1900-066 PMC9004335

[19] Aristidou A Jena R Topol EJ. Bridging the chasm between AI and clinical implementation. Lancet. (2022) 399:620. doi: 10.1016/S0140-6736(22)00235-5, PMID: 35151388

[20] Acosta JN Falcone GJ Rajpurkar P Topol EJ. Multimodal biomedical AI. Nat Med. (2022) 28:1773–84. doi: 10.1038/s41591-022-01981-2 36109635

[21] Sarker IH. Deep learning: a comprehensive overview on techniques, taxonomy, applications and research directions. SN Comput Sci. (2021) 2:420. doi: 10.1007/s42979-021-00815-1 34426802 PMC8372231

[22] Serra A Ruff R Kaminski H Leigh RJ. Factors contributing to failure of neuromuscular transmission in myasthenia gravis and the special case of the extraocular muscles. Ann N Y Acad Sci. (2011) 1233:26–33. doi: 10.1111/j.1749-6632.2011.06123.x, PMID: 21950972

[23] Ouanounou G Baux G Bal T. A novel synaptic plasticity rule explains homeostasis of neuromuscular transmission. eLife. (2016) 5:2190. doi: 10.7554/eLife.12190, PMID: 27138195 PMC4854514

[24] Hoffmann S Ramm J Grittner U Kohler S Siedler J Meisel A. Fatigue in myasthenia gravis: risk factors and impact on quality of life. Brain Behav. (2016) 6:e00538. doi: 10.1002/brb3.538, PMID: 27781147 PMC5064345

[25] Regnault A Morel T de la Loge C Mazerolle F Kaminski HJ Habib AA. Measuring overall severity of myasthenia gravis (MG): evidence for the added value of the MG symptoms PRO. Neurol Ther. (2023) 12:1573–90. doi: 10.1007/s40120-023-00464-x, PMID: 37166675 PMC10444722

[26] van Lummel RC Walgaard S Pijnappels M Elders PJM Garcia-Aymerich J van Dieën JH. Physical performance and physical activity in older adults: associated but separate domains of physical function in old age. PLoS One. (2015) 10:e0144048. doi: 10.1371/journal.pone.0144048, PMID: 26630268 PMC4667847

[27] Dishnica N Vuong A Xiong L Tan S Kovoor J Gupta A. Single count breath test for the evaluation of respiratory function in myasthenia gravis: a systematic review. J Clin Neurosci. (2023) 112:58–63. doi: 10.1016/j.jocn.2023.04.011, PMID: 37094510

[28] Delmondes GMB Couto NFS Correia Junior MGA Bonifácio ABS Freitas Dias R Bezerra J. Single breath counting technique to assess pulmonary function: a systematic review and meta-analysis. J Breath Res. (2023) 18:014001. doi: 10.1088/1752-7163/ad0647 37875103

[29] Rb-Silva R Goios A Kelly C Teixeira P João C Horta A. Definition of immunological non-response to antiretroviral therapy: a systematic review. J Acquir Immune Defic Syndr. (2019) 82:452–61. doi: 10.1097/QAI.0000000000002157, PMID: 31592836

[30] Leigh RJ Zee DS. Mathematical models: an extension of the clinician’s mind. Prog Brain Res. (2019) 248:19–26. doi: 10.1016/bs.pbr.2018.11.001, PMID: 31239131

