# Supplementary material for: Application of digital tools and artificial intelligence in the Myasthenia Gravis Core Examination
Source: Front Neurol. 2024 Dec 4;15:1474884. doi: 10.3389/fneur.2024.1474884 (PMC11652356; doi:10.3389/fneur.2024.1474884)
Supplement: Supplementary file 1 [file Table_1.docx]

**Supplement Methods and Observation**

Garbey et al. Application of Digital Tools and Artificial Intelligence to Optimize the Myasthenia Gravis Core Examination Obtained by Telemedicine

| **SUPPLEMENTAL TABLE 1 Demographics and Clinical Characteristics** | |
| --- | --- |
| **Patients** | **N=52** |
| N (%) women | 26/52 (50%) |
| Age (median), yrs  Min  Max | 63.3  23.5  88.3 |
| N (%) white | 44/52 (84.6%) |
| MGFA severity class  No signs of active disease  Class I  Class IIa  Class IIb  Class IIIa  Class IIIb | 4 (7.7%)  16 (30.8%)  10 (19.2%)  15 (28.8%)  3 (5.8%)  4 (7.7%) |
| **Control Subjects** | **N=12** |
| N (%) women | 7/12 (58%) |
| Age (median)  Min  Max | 55  20  81 |
| N (%) white  Black  Asian | 8/12 (67%)  1/12 (8%)  3/12 (25%) |

**Detailed Methods**

**General Comments**

The number of frames per seconds (fps) and number of pixels for the patient frame are critical metrics for analysis. All Zoom videos were found to run at 25 fps except for five running at 30 fps. The number of pixels allocated to the view of the patient during the examination varied by at least a factor 3 across videos. The distance between the patient and the camera varied across videos adding an addition factor of heterogeneity and potential impacting the assessment. Image contrast, subject illumination and background images varied but had minimal influence on the quality of the data acquisition.

**Ptosis Evaluation**

- Distance Lower Lid from Upper Lid before & after the 60s
- Lid fatigue: Distance lower lid bottom to iris lower boundary and distance lower lid from upper lid during the 60 s look up performance test.
- **Verification:** checking consistency on eyes width and eyes height during the video, sensitivity of output, percentage of the number of frames of the video processed, visual verification of ptosis fatigue on video.

**Diplopia**

The change in Barycentric coordinate of the visible one side iris boundary along the horizontal axis: showing either fatigue (movement of each eye back to center) or squinting. For now, this metric does not compute potential misalignment of eyes in the vertical direction that occurs in few patients for this clinical study.

Distance upper lid to lower lid during the test, as defined in the ptosis fatigue test.

The time stamp when the patient reports they report double vision.

**Verification:** as per Ptosis for the computer vision piece, additional check by comparing the transcript of what the patient said and the video.

**Arm Extension**

Elapse time, up to 2 minutes, that the patient can maintain arm position up during the test.

Potential drift down of the a angle of the arms to thorax during the exercise, approximated by a least square line.

**Verification**

We checked that the algorithm finds the same arm size on each side during the performance test. Inconsistency on these dimensions between markers are characteristics of instability in the identification of anatomic markers. Visual verification on video on elapse time the patients hold his arm up. Visual verification of arm landmarks on a representative sample of video.

**Sit-to-Stand**

Elapsed time of the vertical ascension phase to stand up with or without arm crossed expressed in second

For patient facing the camera: it is possible to measure the deviation of the trajectory of head and shoulders markers from the vertical during the ascending phase: to get a simple metric assessment, we compute the standard deviation of that deviation and normalize it with respect to its vertical dimension. This metric might be indicative of balance issues.

For patient side view: One may decompose the movement into phases and asses their timing separately: it has not been done in this study due to the generally poor conditions of video acquisition with patient at home detailed below – ref [: Li J, Xue Q, Yang S, Han X, Zhang S, Li M, Guo J., Kinematic analysis of the human body during sit-to-stand in healthy young adults. Medicine 2021;100:22(e26208)].

**Verification:**

We compute automatically the percentage of elapse time the head is visible in video during the exercise.

We do the same for the hip.

We used systematically visual verification playing video in slow mode to get verify elapse time of the ascending phase.

**Cheek Puff**

1. Interval of time when the mouth is firmly closed inside the time window of the exercise.
2. Note: markers such as lips deformation depends very much on subject: at this point of our study, it still does not give us a quantitative metric and localization of muscle strength.

**Verification:** Visual verification on a representative sample of video.

**Tongue to Cheek**

Idem as above

**Counting from one to 50: intended to detect Dysarthria**

**Metrics:** we compute the time evolution of the vertical mouth opening during the test, and use the average variation of the acceleration of that vertical mouth distance as a marker of muscle activity.

We computed also a number of standard speech features such as loudness, pitch, pause between numbers, entropy, spectral distribution of signal to build a training set that might be used eventually in pattern recognition as in [ 11-13].

**Verification:** check with transcript that all number are said and clearly pronounced. Check time frequency and time interval between words using NPL.

**Counting on one breath: intended to detect diaphragm muscle weakness.**

**Metrics**

Elapse time of counting and TBA

**Verification:** check with transcript that all number are said and clearly pronounced. Check time frequency and time interval between words using NPL. Visual verification of elapse time playing video.

--------------------------

**Supplemental Table 2. Number of Video discards out of 87 videos of Patient**

| Ptosis | Diplopia Looking Left | Diplopia Looking Right | Arm Extent | Sit to Stand | Lip Motion |
| --- | --- | --- | --- | --- | --- |
| 37 | 40 | 45 | 32 | 65 | 14 |

Remark: In principle the total number of videos should have been 102 in correspondence to 51 patients with 2 visits each. But, for some technical reasons, another 15 videos were missing either because of a missing visit (3) or an inadequate recording with zoom (12).

--------------------------

**Supplemental Table 2. Reasons for Removal of Video from Analysis**

**General**

- Alternating view from examiner view to patient view during test by itself disrupt the possible digital processing of the patient view for 12 videos out of 99 visits.

**Ptosis Test**

- Face not included in the image frame, i.e., patient too close to camera
- Face too far from camera leading to lower resolution.
- Subject holding head back
- Poor light conditions
- Frontalis Activation
- Target subject is looking at is not high enough.

**Diplopia Test**

In addition to Ptosis is test

- Subject’s head turned in gaze direction

**Arm Extension**

- Subject too close to camera (Hand and portion of arm not visible)
- Loose garment hides arm contour, especially portion of arm closest to the ground

**Sit to Stand test**

- Patient too close to camera with either or both
  - Head disappearing during stand up
  - Hip missing during stand up

**Counting to 50 & counting on one breath**

- Volume is too low
- External noise perturbs the record.

**Supplement Figure 1. Duration of One Breath Count**


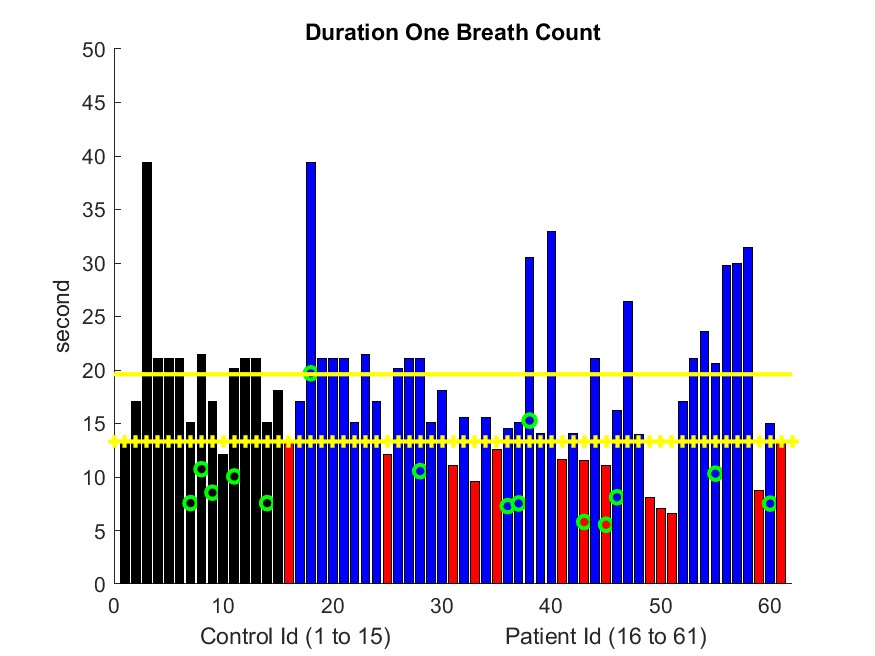


Elapsed time for counting on one breath. Control results represented by the black bar and MG subjects in blue except red bars showing significant differences in time of count. Green circle marks subject older than 70 years. The Yellow horizontal lines represent the mean metric output for control and the mean plus one standard deviation as a threshold to mark the sign of breathing weakness.

**Supplement Figure 2. Linear drift of the arms during extension**


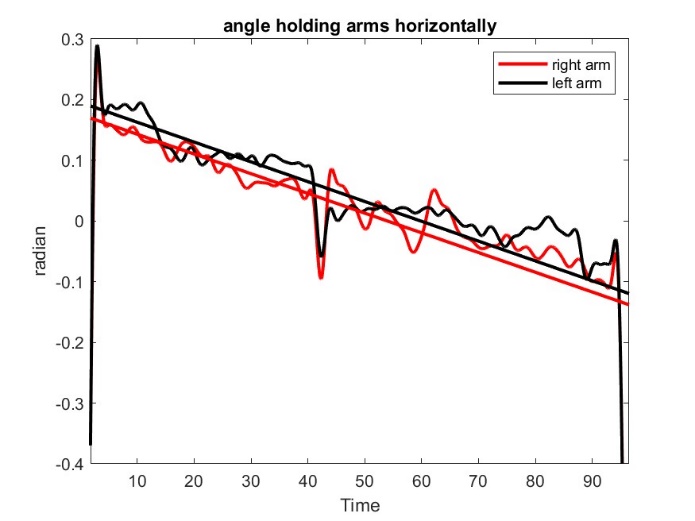

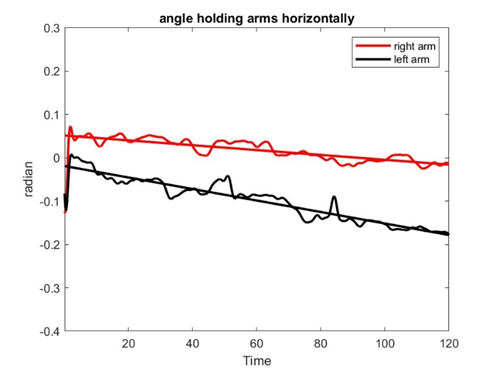


**B**

**A**

Example of the linear drift of arms during arm extension. (A) shows a subject with symmetric drift while (B) identifies asymmetric weakness.
